# Draft genome sequences of 13 putatively novel *Haemophilus* species and strains assembled from human saliva

**DOI:** 10.1128/mra.00945-23

**Published:** 2024-02-20

**Authors:** Daniel Saito, Cristiane Pereira Borges Saito, Fabiana de Souza Cannavan, Siu Mui Tsai

**Affiliations:** 1 Computational Biology Laboratory, Superior School of Health Sciences, Amazonas State University, Manaus, Amazonas, Brazil; 2 Functional Genomics Laboratory, Superior School of Health Sciences, Amazonas State University, Manaus, Amazonas, Brazil; 3 Cellular and Molecular Biology Laboratory, Center for Nuclear Energy in Agriculture, University of São Paulo, São Paulo, Brazil; Montana State University, Bozeman, USA

**Keywords:** *Haemophilus*, saliva, oral, bacteria, metagenome

## Abstract

We present the draft metagenome-assembled genomes (MAGs) of 13 *Haemophilus* representatives from human saliva. MAGs were reconstructed by a streamlined pre-assembly mapping approach performed against 9 clinically relevant reference genomes. Overall, genomes belonging to 2 potentially novel *Haemophilus* species and 11 strains were recovered, as determined by genome-wide ANI analysis.

## ANNOUNCEMENT

*Haemophilus* are small gram-negative cocobacilli that inhabit the oral cavity and upper respiratory tract of humans (1, 2). Under dysbiotic conditions, *Haemophilus* spp. may engender localized and systemic infections, including otitis media, sinusitis, conjunctivitis, pneumonia, chancroid, meningitis, and bacteremia (2). Members of this genus are generally fastidious and difficult to culture in the laboratory (2); hence, molecular-based investigations can help shed additional light into their virulence and pathophysiology. In this study, 13 *Haemophilus* metagenome-assembled genomes (MAGs) were recovered from non-stimulated saliva of healthy and oral disease-associated subjects.

Twenty-seven volunteers were attended at the Dental Clinic of Amazonas State University (Brazil) with no distinction to gender, age, or ethnicity. All participants signed an informed consent complying to the seventh version of the Declaration of Helsinki (2013). Non-stimulated saliva samples were collected and submitted to total DNA extraction. Metagenomic DNA was hydrodynamically sonicated, and 1.0 µg DNA from each sample was used for preparation of sample-specific libraries with NEBNext Ultra DNA Library Prep Kit. Products in the range of 300 bp were selected and sequenced via Illumina HiSeq 2500 platform. Paired-end reads were merged and adapter sequences excised with PEAR v.0.9.8, while host-related sequences were removed by mapping to the GRCh38.p14 human genome data set (https://www.ncbi.nlm.nih.gov/datasets/genome/GCF_000001405.40, accessed on 02 June 2023) with Bowtie2 (3), using the “–un-conc” and “--very-sensitive-local” parameters. Species-level mapping of reads was performed with Bowtie2 (3) using the “--fast-local” parameter against NCBI’s reference genomes of *H. aegyptius*, *H. ducreyi*, *H. haemolyticus*, *H. influenzae*, *H. parainfluenzae*, *H. parahaemolyticus*, *H. paraphrohaemolyticus*, *H. pittmaniae,* and *H. sputorum*. Assembly of contigs and binning of MAGs were achieved with SPADES v.3.15.5 (4) and Maxbin2, respectively. Completeness (50% minimum) and contamination (10% maximum) values were assessed with CheckM v.1.10.18 (5). Taxonomic placement of MAGs was achieved with GTDB-Tk v1.7.0 (6) of Kbase (7), adopting ANI scores of 95% for species-level definition and between 95% and 97% for strain-level demarcation (8). General MAG annotation was performed with NCBI’s PGAP v.4.11. Search for antimicrobial resistance determinants was performed with CARD 2023 (9) and annotation of carbohydrate-active enzymes, with dbCAN3 (10).

In all, 113 MAGs were binned from a total of 27 clinical samples. Of these, 13 MAGs fully complied to the quality and taxonomic threshold parameters and were, therefore, further selected for taxonomic inference and gene annotation procedures. These MAGs were recovered from 11 distinct saliva specimens, with sample OHS0020_HPI being related to a chronic periodontitis case, and the remainder to healthy subjects. According to GTDB-tk analysis, all MAGs were placed within the *Haemophilus* genus, 11 of which corresponding to previously unreported strains of *H. haemolyticus*, *H. parahaemolyticus,* and *H. parainfluenzae*. In addition, MAGs OH0009_HAE and OH0010_HAE displayed ANI values <95% with those available in GTDB, suggesting them as putatively novel genomes closely related to *H. parainfluenzae*. These were deposited in Genbank under names *Haemophilus* bacterium OH0009 and OH0010, respectively. General taxonomic and annotation features are depicted in Table 1.

**TABLE 1:** General information and annotation results of 13 metagenome-assembled genomes belonging to the *Haemophilus* genus retrieved from non-stimulated saliva of 11 individuals

Characteristic	Data for MAG				
	OHS002_HPH	OHS0003_HPI	OHS004_HHA	OHS0004_HPI	OHS0005_HPI
Species-level taxonomy	*H. parahaemolyticus*	*H. parainfluenzae*	*H. haemolyticus*	*H. parainfluenzae*	*H. parainfluenzae*
Host clinical condition	Oral health	Oral health	Oral health	Oral health	Oral health
SRA accession no.	SRR14122749	SRR14122738	SRR14122730	SRR14122730	SRR14122729
Biosample accession no.	SAMN34352703	SAMN34352705	SAMN34352701	SAMN34352706	SAMN34352707
Genbank accession no.	JAUPSI000000000	JAUPSK000000000	JAUPSG000000000	JAUPSL000000000	JAUPSM000000000
N50 (bp)	12,080	1,944	4,297	3,889	3,178
Genome size (nt)	1,847,273	1,130,633	1,266,290	1,640,887	1,537,644
Genome completeness	97.84	68.84	77.93	92.06	86.26
Genome contamination	2.01	3.98	2.89	8.54	6.25
Genome assembly coverage	16.96	9.56	10.59	22.11	24.51
GC content (%)	40.45	39.86	38.85	39.85	39.81
Protein-encoding genes	1,823	1,503	1,322	1,879	1,846
RNA genes	45	21	18	35	41
tRNA genes	40	18	16	29	36
ncRNA genes	4	3	2	4	4
Pseudogenes	26	16	15	13	8
Antibiotic resistance genes	118	87	86	119	101
Carbohydrate-active enzymes	80	50	54	71	59

	OHS0006_HPI	OHS0009_HAE	OHS0010_HAE	OHS0010_HPH	OHS0011_HPI
Species-level taxonomy	*H. parainfluenzae*	*H. bacterium*	*H. bacterium*	*H. parahaemolyticus*	*H. parainfluenzae*
Host clinical condition	Oral health	Oral health	Oral health	Oral health	Oral health
SRA accession no.	SRR14122728	SRR14122725	SRR14122724	SRR14122724	SRR14122748
Biosample accession no.	SAMN34352708	SAMN34352699	SAMN34352700	SAMN34352702	SAMN34352709
Genbank accession no.	JAUPSN000000000	JAUPSE000000000	JAUPSF000000000	JAUPSH000000000	JAUPSO000000000
N50 (bp)	1,705	1,777	3,529	2,386	1,748
Genome size (nt)	945,495	1,016,019	727,606	1,220,189	1,002,532
Genome completeness	62.8	61.7	51.17	69.89	59.14
Genome contamination	3.62	3.25	0.52	1.77	3.02
Genome assembly coverage	14.46	12.88	83.2	5.41	25.19
GC content (%)	39.92	40.06	39.74	40.94	39.85
Protein-encoding genes	1,298	1,366	888	1,369	1,339
RNA genes	28	9	28	19	25
tRNA genes	26	7	24	14	21
ncRNA genes	1	2	3	4	4
Pseudogenes	12	12	2	48	8
Antibiotic resistance genes	70	83	48	89	75
Carbohydrate-active enzymes	47	41	23	54	45

	OHS0012_HPI	OHS0013_HPH	OPS0020_HPI		
Species-level taxonomy	*H. parainfluenzae*	*H. parahaemolyticus*	*H. parainfluenzae*		
Host clinical condition	Oral health	Oral health	Chronic periodontitis		
SRA accession no.	SRR14122747	SRR14122746	SRR14122739		
Biosample accession no.	SAMN34352710	SAMN34352704	SAMN34352711		
Genbank accession no.	JAUPSP000000000	JAUPSJ000000000	JAUPSQ000000000		
N50 (bp)	1,730	1,927	1,831		
Genome size (nt)	967,926	1,057,189	1,205,798		
Genome completeness	59.49	58.08	73.6		
Genome contamination	6.26	2.18	6.29		
Genome assembly coverage	20.15	5.87	12.55		
GC content (%)	39.69	40.38	39.80		
Protein-encoding genes	1,326	1,341	1,605		
RNA genes	16	26	37		
tRNA genes	11	23	31		
ncRNA genes	3	2	4		
Pseudogenes	7	31	12		
Antibiotic resistance genes	60	68	95		
Carbohydrate-active enzymes	45	44	46		

## Data Availability

Raw reads and biosamples belong to NCBI’s bioproject no. PRJNA717815 are available under the accession numbers displayed in Table 1.
